# Prediction of difficult intubations using conventional indicators: Does rapid sequence intubation ease difficult intubations? A prospective randomised study in a tertiary care teaching hospital

**DOI:** 10.4103/0974-2700.76836

**Published:** 2011

**Authors:** Lakshmi Gangadharan, C Sreekanth, Mabel C Vasnaik

**Affiliations:** Department of Emergency Medicine, St. Johns’ Medical College and Hospital, Bangalore, India

**Keywords:** Airway assessment, conventional indicators, difficult intubation, emergency department, Mallampatti score, rapid sequence intubation

## Abstract

**Background:**

Endotracheal intubations performed in the Emergency Department.

**Aims:**

To assess whether conventional indicators of difficult airway can predict a difficult intubation in the Emergency Setting and to investigate the effect of rapid sequence intubation (RSI) on ease of intubation.

**Settings and Design:**

A prospective randomized study was designed involving 60 patients requiring intubation, over a period of 4 months.

**Materials and Methods:**

Demographic profile, details of methods used, airway assessment, ease of intubation, and Cormack and Lehane score were recorded. Airway assessment score and ease of intubation criteria were devised and assessed.

**Statistical Analysis:**

Descriptive statistical analysis was carried out. Chi-square/2 × 2, 2 × 3, 3 × 3, Fisher Exact test have been used to find the significance of study parameters on categorical scale between two or more groups.

**Results:**

Patients with a Mallampatti score of three or four were found to have worse laryngoscopic views (Cormack–Lehane score, 3 or 4). Of all airway indicators assessed, an increased Mallampatti score was found to have significant correlation with increased difficulty in intubation. The use of RSI was associated with better laryngoscopic views, and easier intubations.

**Conclusions:**

An airway assessment using the Mallampatti score is invaluable as a tool to predict a difficult airway and should be performed routinely if possible. RSI aids intubation ease. If not otherwise contraindicated, it should be performed routinely for all intubations in the ED.

## INTRODUCTION

The ability to manage the airway of critically ill patients is an essential skill in the practice of emergency medicine. Most patients have a full stomach or an unknown fasting status and have had no premedication. It is well documented that airway management in the busy emergency department (ED) is at opposite spectrums from the controlled environments of the operating room. The need for tracheal intubation in the ED is usually unpredictable and often promptly required.[1]

Data regarding difficult intubations in the ED are few although some reviews have stated that in the operating room (OR), the incidence of difficult intubation is 1.15–3.18% and in the ED it is 3–5.3%.[2,3] Only 0.01% of patients intubated electively will have airway management failure, as compared with an ED rescue cricothyrotomy rate of 1%.[2] It has been suggested that patients intubated in the ED have poorer views on laryngoscopy measured by higher Cormack and Lehane scores.[4,5] It can thus be construed that intubations in the ED may be more difficult to perform than those taking place in the OR. The ED physician should also consider the fact that not all difficult airway indicators can be assessed in every patient presenting to the ED and he/she should adapt his/her assessment to a wide variety of scenarios in the ED.

“Difficult airway” is one in which there is a problem in establishing or maintaining gas exchange via a mask, an artificial airway or both. Intubation is considered difficult if more than three attempts are necessary or if conventional laryngoscopy requires more than 10 min. It represents a complex interaction between patient factors, the clinical setting and the skills of the practitioner.[4]

The identification of a potentially difficult airway is perhaps more important than the subsequent management and may obviate a rescue airway in the “cannot ventilate–cannot intubate” scenario. Before any attempt at airway management, an assessment of potential difficulties must be performed. Once the potential for difficulty is identified, the management will vary with not only the type of airway difficulty, but also with the operator’s experience and the availability of alternative devices.

Airway assessment scales vary from the simple, which often fail to address the many factors associated with a difficult airway, to the complex, which are impractical as a clinical tool in a busy ED. An assessment system that objectively measures factors associated with a difficult intubation needs to be simple to perform, suitable for unconscious or uncooperative patients, and easily remembered. All of these criteria are rarely satisfied in the ED setting.[1]

Rapid sequence intubation (RSI) is widely accepted as the method of choice in ED intubations. Used judiciously, its advantages far outweigh its limitations.

About 70% of the ER patients who undergo RSI have several factors, which prevent the assessment of these predictive factors. When RSI is used appropriately in the ED, it increases the success rate of intubation to 98% while reducing complications.[6]

Presence of a definitive difficult airway program in the ED helps mitigate the need for emergency surgical airway procedures whenever unable to intubate and ventilate.[7]

Although studies on difficult intubation and its indicators are in abundance, most tend to disregard the utility of the Mallampati score in the ED. Unavailability of information regarding emergency intubations in an Indian ethnic population was also a limiting factor. This study aimed to fill the lacuna in that field, by finding out if conventional airway indicators could accurately predict a difficult intubation, and also assess if the use of RSI led to easier intubations, as compared to those done without the aid of paralytic agents.

## MATERIALS AND METHODS

This was a prospective observational study conducted in the adult ED of a tertiary care teaching hospital. The study was designed to formulate a protocol for difficult airway assessment to be used routinely in our ED. Sixty patients were assessed over a period of 4 months. All patients above the age of 15 years who required emergency intubation were included, and those patients requiring a primary surgical airway were excluded from the study. Approval from the Institutional ethics committee review board was obtained. The demographic profile of the patients was recorded, and if time permitted, the patients were assessed by the airway manager and scored for neck extension, Mallampatti score, mouth opening, thyromental distance, and whether airway obstruction, facial trauma, or anatomic barriers were present. The original Mallampati score was used.[8] A partial view (*n* = 21) or no view (*n* = 2) was considered a predictor of difficult intubation.

The Mallampatti score was assessed by asking the patient to sit upright, open the mouth as wide as possible, with maximum tongue protrusion and no phonation.

The Revised Mallampatti and Extended Mallampatti score, though possibly better modes of assessment and statistical significance, was considered by the authors as impractical and time consuming in the Emergency setting. Neck extension was either normal, with full range of movements, or reduced. Mouth opening or interincisor distance was measured by the operators’ fingers and graded as 3, 2, 1, or no fingers. Less than or equal to two fingers was taken as a predictor of difficult intubation. Thyromental distance was also assessed using fingerbreadths of the operator. Less than three fingers were taken to be an indicator of difficult intubation.

In cases where time was a critical factor, or the patient was unresponsive or uncooperative, the airway assessment was either not done or done partially. These patients, nevertheless, were included in this study to assess the feasibility of performing airway assessment in the emergency setting. The airway management of the patients were never compromised for the sake of this study. All adverse events in the immediate postintubation period were specified.

**Figure 1:**
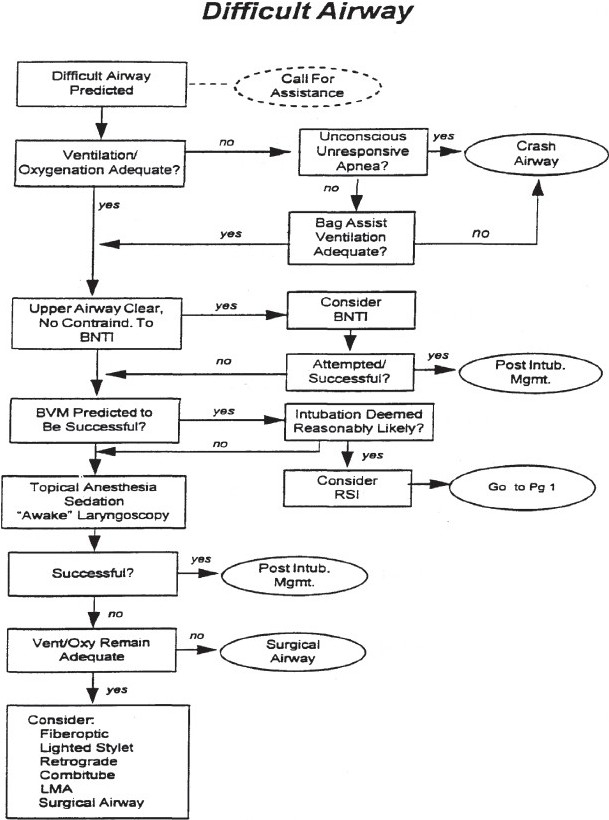
Adapted from the ACEP Difficult Airway course^®^ 2007

Intubation was performed in the ED using standard methods and preferably using RSI technique to render the patient unconscious and paralyzed within 1 min. Monitors used included an ECG monitor, noninvasive blood pressure monitor, and a pulse oximeter. Adequate preoxygenation was provided to all patients. Induction was done with Propofol (1–2 mg/kg)/Ketamine (1–2 mg/kg)/Thiopentone sodium (2–5 mg/kg) or Midazolam (0.1 mg/kg) given intravenously. Paralytic agents used include Rocuronium (0.8–1.2 mg/kg)/Vecuronium (0.08–0.1 mg/kg) or Succinylcholine (1.0–1.5 mg/kg) of which Succinylcholine was the markedly preferred drug due to shorter duration of action, and better performance. The Mackintosh blade (size three or four), appropriately sized ET tubes and bougie were also used in some cases to aid difficult intubations. Airway adjuncts kit was always kept as standby in the case of a failed intubation. The following Difficult Intubation Algorithm [Figure 1] devised by the American College of Emergency Physicians (ACEP) was used in all predicted difficult intubations of which early recognition of a difficult intubation was the most emphasized step since the only way to fall on to the Difficult Airway Algorithm is from the box, “Predict Difficult Airway?” on the Emergency Airway Management Algorithm. Thus, airway assessment becomes a vital skill.

The airway manager, who intubated the patient and graded the ease of intubation, was different from the operator who assessed the airway and measured the vitals. This was done to prevent bias. The call for assistance, once difficult airway was identified, went only to senior ED physicians with more than 2 years experience in airway management in ED. The Airway Adjuncts Kit contained a combitube, a trans-tracheal needle ventilation device, an intubating LMA, flex-guide introducer (bougie), tube exchangers, and a surgical cricothyroidotomy set. The fibreoptic bronchoscope, although available, was not utilized, since operator expertise, as well as time, was critical-limiting factors.

A Cormack and Lehane view of 3 or 4, increased lifting force, BURP maneuver used, inability of a senior airway manager to intubate in two attempts, and vocal cords adducted/closed on laryngoscopy were classified as a difficult intubation. A printed data entry form was used to record all information pertinent to this study.

### Data collection and statistical analysis

Descriptive statistical analysis has been carried out in this study. Results on continuous measurements are presented on Mean ± SD (min–max) and results on categorical measurements are presented in number (%). Significance is assessed at 5% level of significance. Chi-square/2 × 2, 2 × 3, 3 × 3, Fisher Exact test have been used to find the significance of study parameters on categorical scale between two or more groups. The 95% confidence interval has been computed to find the significant features. Confidence interval with lower limit more than 50% is associated with statistical significance. A ’*P*’ value of less than 0.05 was considered as statistically significant. The statistical softwares: Statistical Package for the Social Sciences (SPSS) software for Windows (version 15; SPSS Inc.), Stata 8.0, MedCalc 9.0.1, and Systat 11.0 were used for the analysis of the data.

## RESULTS

Our study demonstrated a predominance of the male patients aged between 51 and 75 years who were intubated. The most common indications for intubation, which included head injury (40%), followed by cerebrovascular accident (18.3%), pneumonia (13.3%), drug overdose (10%), and shock (10%).

### Results pertaining to difficult intubation and its indicators

We were able to intubate 97% of the patients in the first two attempts. Only 3.3% of patients needed a third attempt for successful intubation. There was no failed airway or mortality due to the same in the ED during the course of this study. The most frequent method of intubation used was RSI (46.7%). Fifteen percent of patients were intubated without any medications. The remaining 38.3% were sedated, but not paralyzed. The laryngoscope alone was enough for a majority of intubations (91.7%), and the remaining were aided by the flex guide (8.3%). The most common complications encountered in the immediate postintubation period were direct airway injury (6.7%), dental trauma, and esophageal intubations (6.7%). We experienced intubation difficulty in 35% of intubations. A Cormack and Lehane grade 3 glottic visualization was seen only in 8.3% of intubations. There was a positive correlation between better Mallampatti scores and actual ease of intubation (*P* = 0.004). Correlation between other predictors of difficult intubation and actual ease of intubation failed to reach significance. Correlation between difficult intubation as per Cormack and Lehane glottic exposure score and predictors of difficult intubation was found not to be of any statistical significance.

### Results pertaining to RSI and difficult intubation

There was a significant correlation between the use of RSI and better glottic visualization (*P* = 0.005) but not for the ease of intubation (*P* = 0.129), as compared to intubations performed with sedation only or when no medications were used. Visualization of cords was better with RSI (85% visualized entire cords), as compared to without use of paralytics (50%). Intubations were easier while using RSI (75%) as compared to without use of RSI (56%).

## DISCUSSION

The ED is a place where doctors from various branches of medicine, with different qualifications and designations work. In a country like India where Emergency Medicine is still in its infancy, ease of intubation is achieved with practice. Hence, difficult airways may be identified faster and predicted easily if a readily identifiable group of predictors are listed. The ASA has listed several airway predictors which include length of upper incisors, interincisor distance <3 cm, a Mallampatti class greater than II, highly arched palate, thyromental distance <3 ordinary finger breadths, etc. None of these predictors have been shown to accurately predict the difficulty in airway management. Prediction of a difficult airway is the best performed by assessment of multiple features.[9] The prediction of ease/difficulty of intubation remains an imperfect science since the tests fail to predict some difficult intubations and many predicted intubations turn out to be easy.[10] Conventionally, evaluation of the difficult airway include the LEMON scoring as well as newer assessments such as the height-to-thyrosternal distance ratio and the upper lip bite test. Few studies have achieved significant predictive ability of individual or combination of anatomical variables for difficult airway management.[10] The original Mallampatti test was used to predict difficult tracheal intubation in five studies enrolling a total 12,351 patients. The prevalence of difficult intubations ranged from 6% to 13%. There were low sensitivities and varying specificities.[11] The original Mallampatti test identified difficult intubations with a high degree of accuracy, with sensitivity of 50% and specificity of 100%.[8] Our study demonstrated similar results (*P* = 0.004). The Mallampatti scoring will be of use in the emergency for the evaluation of mentally alert patients who require elective intubation. However, it will be of limited use in critically ill patients or those in respiratory failure who are unlikely to co-operate with the evaluation.[7] Bair and others suggest that clinical instability and lack of patient co-operation prevent the use of the original Mallampatti score in the ED.[12] Such difficulties in assessment may be overcome to some extent by using indicators such as neck extension, thyromental distance, height-to-thyrosternal distance ratio, and assessment of other externally visible parameters such as short neck, buck teeth, small chin, etc. Some recent studies also describe the thyromental distance as a better evaluator of a difficult airway, rather than the Mallampatti score, but our findings were contrary to this.[13] Comparison of difficult intubations between operators and institutes has been difficult. Laryngoscopic view, which is technique dependant and varies among operators, is only one of the several determinants of difficult intubation. Hastings *et al*. demonstrated that the laryngoscopic force, torque, and head extension depend on the technique, expertise, and equipment. A greater force might be required because of anatomic reasons, such as decreased compliance or mobility of the pharyngeal tissues, or the force might result from increased effort in the face of a poor view.[14] RSI refers to the nearly simultaneous administration of an induction agent with a neuromuscular blocker for emergency airway management. If it is used appropriately by trained/skilled operators, the success rate of intubations can be increased to 98% while reducing the complications.[15–17] Emergency physicians have modified the term rapid sequence induction to rapid sequence intubation. This subtle difference in semantics is meant to represent a different end-point: in the operating room, patients are intubated to provide anesthesia while in the ED patients are anesthetized/paralyzed to facilitate intubation.[16] Taryle *et al*. showed that ED intubations without neuromuscular blockade could have significant complications with high rates of prolonged intubation and aspiration. Administration of these agents by nonanesthesiologists, however, is controversial in some centers.[15] Recent articles using RSI in the ED have studied larger populations and shown little risk of adverse events.[18] Kovacs and others recommend that RSI must be used commonly in the ED and cite a number of airway courses (Airway course^®^, Airway Interventions^®^, and Management Education Program^®^) which suggest RSI be used as the default method of intubation unless a contraindication precludes it.[16] Studies reviewed by the EAST workgroup indicate that there exists a substantial experience with emergency drug-assisted intubation in trauma patients in multiple settings and that the intubation success-rate approaches, but does not reach 100%.[19] However, RSI is not without risk, particularly if the airway cannot be readily secured and adequate ventilation cannot be provided.[17] Sugammadex^®^, the reversal drug for Rocuronium, was not easily available and the cost was a limiting factor for its routine use in an Indian ED. For this reason, we used Succinylcholine most commonly during intubations, due to its shorter duration of action, unless absolutely contraindicated. Reynolds and Heffner recommend that in the presence of severe acidosis, intravascular volume depletion, cardiac decompensation, and severe lung injury, crash intubation is preferred since the patients have a depressed consciousness and tolerate intubation attempts.[6] This is contrary to our study wherein 46% of our patients were intubated with RSI and the remaining with sedatives, or no drugs. Considering the groups of patients presenting to our ED and the instability they have, this would be the appropriate method of airway management.

### Study limitations

A larger population study is vital to validate results. This study, which was conceived as a pilot project, attempts to direct the course of a larger work, and also hopes to fill the lacunae which currently exist where airway management in Indian EDs are concerned. Factors such as duration of laryngoscopy attempts, the number, and seniority of airway managers involved, though described as markers of difficult intubation, have not been included in this study because of their many confounding factors. These may, however be assessed in further studies. Time available for airway assessment was a critical issue in this study. Almost 33% of patients did not have a complete or partial assessment of their airway, before being intubated. Inadequate dosing of premedication, induction agents, and/or paralytic drugs, under reporting of post intubation events by the airway manager were all other possible limiting factors.

## CONCLUSION

An airway assessment using the Mallampatti score is invaluable as a tool to predict a difficult airway and we suggest that the Mallampatti score was performed in addition to and as an integral part of routine assessment of difficult airway indicators in the ED.

RSI aids intubation ease. If not otherwise contraindicated, it should be performed routinely for all intubations in the ED.

In conclusion, if difficult airway was predicted early, then more senior help with advanced or alternative airway techniques should be sought, ultimately benefitting the patient.
